# Supplementary material for: Physiological Responses to Salt Stress at the Seedling Stage in Wild (Oryza rufipogon Griff.) and Cultivated (Oryza sativa L.) Rice
Source: Plants (Basel). 2024 Jan 26;13(3):369. doi: 10.3390/plants13030369 (PMC10857172; doi:10.3390/plants13030369)
Supplement: Supplementary file 1 [file plants-13-00369-s001.zip › Supplementary figures.pdf]

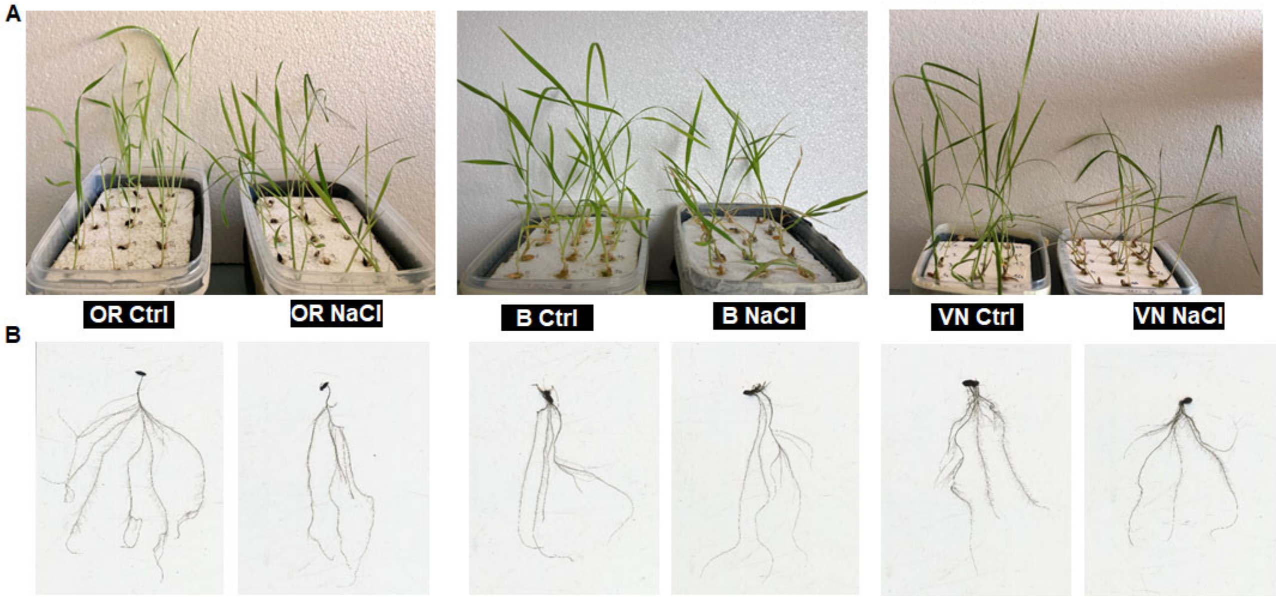

Figure S1. Visual symptoms of salt stress injuries at shoot and root level. Visual symptoms of salt stress injuries after 7 days of exposure to 80 mM NaCl (NaCl) on shoots (**A**) and roots (**B**) of *O. rufipogon* (OR), *O. sativa* var. Baldo (B) and *O. sativa* var. Vialone Nano (VN) with respect to their counterparts from plants grown in control conditions (Ctrl).

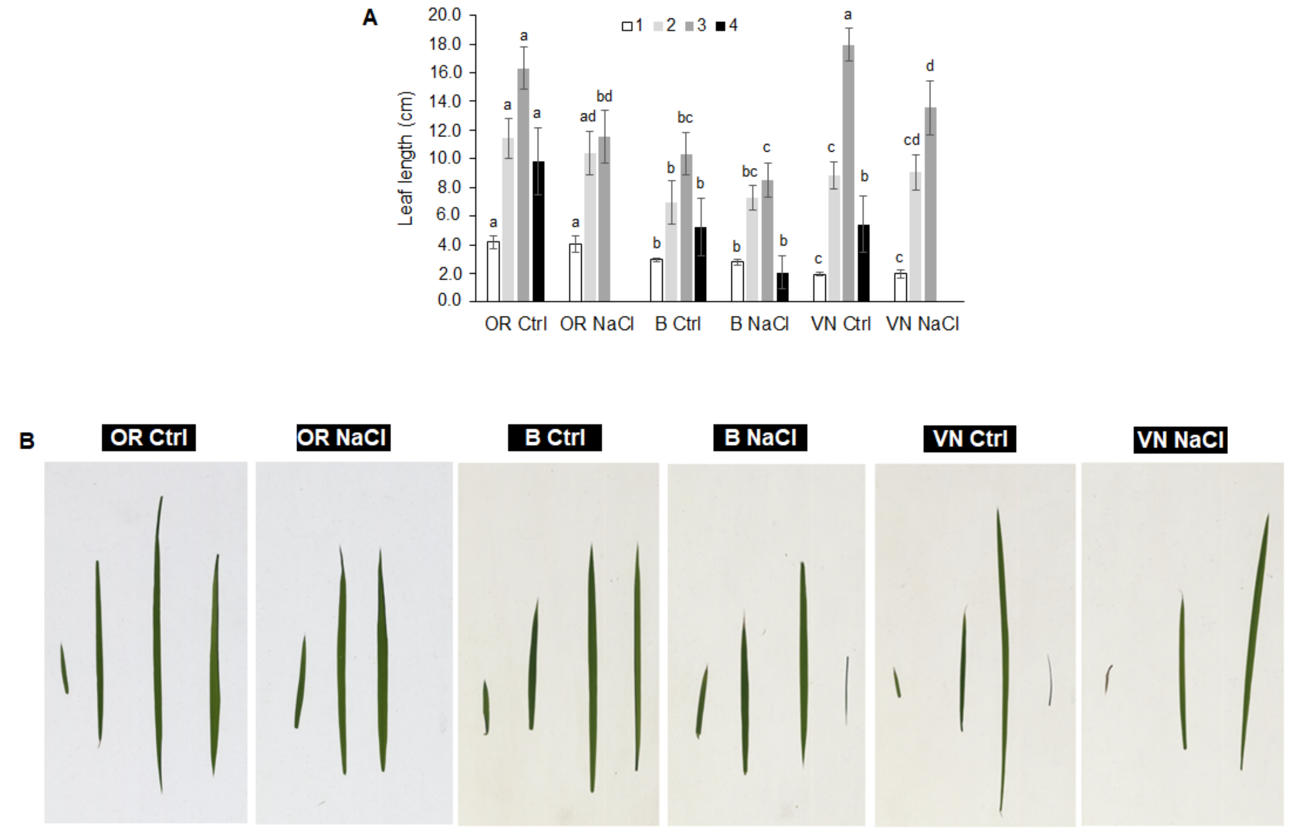

Figure S2. Effect of salt stress on leaf length (1, first; 2, second; 3, third; 4, fourth leaf) evaluated in *O. rufipogon* (OR), *O. sativa* var. Baldo (B) and *O. sativa* var. Vialone Nano (VN) after 7 days of exposure to 80 mM NaCl (NaCl) with respect to their counterparts from plants grown in control conditions (Ctrl) (**A**) and corresponding pictures (**B**). In panel B, leaves are in the order from the first to the fourth starting from the left to the right. Data are the means ± standard deviations of at least eight replicates. For each trait, different letters indicate significant difference determined by One-Way ANOVA with Tukey's test ($p < 0.05$).
